# Optimization of Bead Geometry during Tungsten Inert Gas Welding Using Grey Relational and Finite Element Analysis

**DOI:** 10.3390/ma16103732

**Published:** 2023-05-15

**Authors:** Muhammad Hanif, Abdul Hakim Shah, Imran Shah, Jabir Mumtaz

**Affiliations:** 1 Schools of Mechanical Science and Engineering, Huazhong University of Science and Technology, Wuhan 430074, China; engr.hanif94@yahoo.com; 2 Department of Physics and Chemistry, Khushal Khan Khattak University Karak, Karak 27400, Pakistan; dr.abdulhakim@kkkuk.edu.cn; 3 Department of Aerospace Engineering, College of Aeronautical Engineering, National University of Sciences and Technology, Risalpur 24090, Pakistan; imranshahswabi@gmail.com; 4 College of Mechanical and Electrical Engineering, Wenzhou University, Wenzhou 325035, China

**Keywords:** TIG welding, finite element method, grey relational analysis, thermal stress, mild steel

## Abstract

Mild steel welded products are widely used for their excellent ductility. Tungsten inert gas (TIG) welding is a high-quality, pollution-free welding process suitable for a base part thickness greater than 3 mm. Fabricating mild steel products with an optimized welding process, material properties, and parameters is important to achieve better weld quality and minimum stresses/distortion. This study uses the finite element method to analyze the temperature and thermal stress fields during TIG welding for optimum bead geometry. The bead geometry was optimized using grey relational analysis by considering the flow rate, welding current, and gap distance. The welding current was the most influential factor affecting the performance measures, followed by the gas flow rate. The effect of welding parameters, such as welding voltage, efficiency, and speed on the temperature field and thermal stress were also numerically investigated. The maximum temperature and thermal stress induced in the weld part were 2083.63 °C and 424 MPa, respectively, for the given heat flux of 0.62 × 10^6^ W/m^2^. Results showed that the temperature increases with the voltage and efficiency of the weld joint but decreases with an increase in welding speed.

## 1. Introduction

Mild steel is a type of low-carbon steel that is malleable and ductile, making it ideal for applications that require a low strength-to-weight ratio, such as cages, frames, and fencing. According to Silva et al. (2018), mild steel is preferred over other steels because it endures high stresses and results in longer service life [1]. Due to its malleability, it can be quickly shaped, drilled, welded, and cut. Welding is the fabrication and repair of metal products and an essential process in every industry. It is a simple, cost-effective, and dependable method for joining metals. In welding processes, weld joint quality is usually affected by the weld material’s attributes, mechanical properties, and the highly concentrated localized zone. Saha et al. (2017) stated that the weldability of a material depends upon the properties changing during the welding process and the strength of the centralized weld zone [2]. Datta et al. (2008) noted that the weld functionality features also depend on weld joint geometry, which is directly influenced by the input parameters [3].

Several other welding processes have been proposed to join the specific types and dimensions of work parts: laser welding, TIG welding, electron beam welding, etc. [4]. Cary et al. noted that TIG welding is extensively used in industries because of high weld quality, protection of the weld pool by using an inert gas shield, its versatility, repairability, and flexibility to adapt. Research showed that an optimal welding speed could produce a joint with high mechanical strength, excellent microstructure, and good morphology [5]. Eshwar et al. (2014) found that the input parameter has a substantial role in determining the weld quality, which primarily includes bead width and height, and penetrating the area, thermal, and residual stresses, resulting in minimizing the life of a weld joint [6]. Researchers have tried to work on different welding processes regarding weld bead geometry, temperature, and stress fields. For instance, Dhas et al. (2007) have proposed an adaptive neuro-fuzzy inference system (ANFIS) for determining the optimum input parameters for bead width [7]. A multi-objective optimization of process parameters was carried out to find the optimal bead geometry using the Taguchi method [8]. Fernando et al. (2021) investigated the impact of metal transfer modes on the symmetry of bead geometry. They measured bead geometry using a 3D scanner and found that the metal transfer mode affected the bead geometry symmetry. It provides insight into the effect of metal transfer modes on the quality of WAAM aluminum parts [9]. Improvements in weld quality have been made by combining grey relational analysis with the Taguchi method. Chandrasekhar et al. (2015) have developed an artificial neural network (ANN) to estimate the weld bead width and depth of penetration by using IR images during TIG welding of 6 mm thick 316 LN stainless steel by changing the values of the welding current [10]. Chokkalingham et al. (2012) have also developed a similar model for better estimation of bead width and depth of penetration [11]. Kolahan et al. (2010) established a simulated annealing methodology to analyze and improve the geometry of the weld joint (bead width, bead height, and penetration) in gas metal arc welding [12]. Zhang et al. (2017) have developed an online system for the quality of aluminum alloy welding, which automatically evaluates the quality parameters in real-time using online arc sound, voltage, and spectrum signals [13]. The microstructure, hardness, and corrosion behavior of gas tungsten arc welding (GTAW) Inconel 625 super alloy over A517 carbon steel was also investigated using ERNiCrMo3 filler metal [14]. Bandhu et al. (2022) have experimentally studied the low alloy steel after heat treatment [15]. It was concluded that regulated metal deposition weldments have better mechanical properties than gas welding for the same steel alloy. Jiang et al. (2017) have addressed the quality issues (defects) in the welding process [16]. They proposed a novel approach for classifying weld defects, such as porosity, slag inclusion, lack of penetration, etc., based on the Dumpster–Shafer evidence theory.

In recent years, the main problem faced by researchers is optimizing the factors that affect the welding joint [17] and the geometry of the welding area to predict the temperature distribution, minimum residual stresses, and distortion [18]. Understanding the thermal flow in welding is theoretically and experimentally important. The impact of stress relief heat treatment on the microstructure and mechanical properties of dissimilar GTAW weld joints between Inconel 625 and A106 carbon steel was also investigated [19]. The results provide insights into the effects of heat treatment on the properties of such weld joints. Sepe et al. (2014) demonstrated the residual stresses and temperature distribution in metal arc welding using FEM analysis, finding higher thermal stress in heat-affected and fusion zones [20]. The stresses decrease with the increase in temperature and pre-heating and post-heating methods. Katherasan et al. (2014) performed a simulation study for bead geometry and demonstrated the effects of process parameters such as feed rate, voltage, speed, and torch angle on bead width, reinforcement, and penetration depth using particle swarm optimization (PSO) for flux-cored arc welding [21]. The effect of different process parameters such as voltage, welding current, and wire feed rate on the bead geometry and microstructure of WAAM parts were studied and the results showed that the process parameters significantly impacted the bead geometry and microstructure. It provides insights into optimizing WAAM process parameters for improved part quality and can help guide the WAAM process design [22]. El-Sayed et al. (2017) also predicted the residual stresses and temperature-induced in friction stir welding using finite element analysis [23].

A new calibration coefficient was computed for aluminum plates subjected to uniaxial load. An appropriate coefficient and residual stress compensation value could result in in-situ thermal stresses with expected accuracy within acceptable ranges of the industry’s specification limits [24]. Kulkarni et al. (2019) demonstrated TIG welding joining dissimilar metals for stainless steel (P91 steel and AISI 3161) with Inconel 800 and Inconel 600 [25]. It was observed that an Inconel 600 interlayer and a fully austenitic structure could be achieved without any mechanical loss. Based on the above literature, it has been realized that very little or no work has been reported to study the thermal and structural analysis and variations in temperature with the TIG welding parameters for the optimized process.

This research primarily focuses on analyzing multi-objective optimization of the TIG welding process for an optimum factor selection affecting bead geometry, temperature, and thermal stresses. Taguchi-based grey relational analysis for multiple objectives was employed to determine significant factors affecting the performance measures. The temperature variations over time and thermal stresses for the optimum geometry were studied. ANSYS APDL was used to generate the model, and finite element analysis (FEA) was employed to predict the responses. Heat flux was also determined by conducting the experiments with the selected levels of factors, which was further used in FEA in the V-shaped butt joint. As per the required dimensions, a 3D solid element with DOF, temperature (SOLID-70), and a 2D solid element with four nodes (PLANE-55) was used. A transient analysis followed by the coupled field analysis was performed to predict temperature variations and thermal stresses. The variation of temperature by welding parameters was also investigated.

## 2. Materials and Methods

TIG welding was achieved on the mild steel (MS) plates, and its chemical composition is presented in Table 1 [26]. A CNC milling machine was used to prepare test samples of dimensions 80 × 20 × 3 mm^3^ for the V-shaped butt joint. The samples were appropriately clamped at the ends during welding. In experiments, argon gas was used because of its inert behavior, protecting the molten metal from impurities in the atmosphere and being heavier than air. The joining of metals was performed by melting the work partly with an arc fixed between the electrode (tungsten) diameter of 3.2 mm and the base part. A high-frequency spark delivered a conductive pathway through the air.

**Figure 1:**
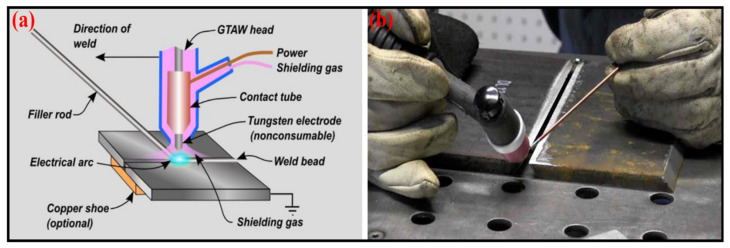
TIG Welding mechanism. (**a**) illustration of TIG welding process (**b**) experimental setup.

Due to the spark produced, the surface of the base work partly melted, and a molten pool was formed. Figure 1 shows a schematic diagram and setup of TIG welding [27].

**Figure 2:**
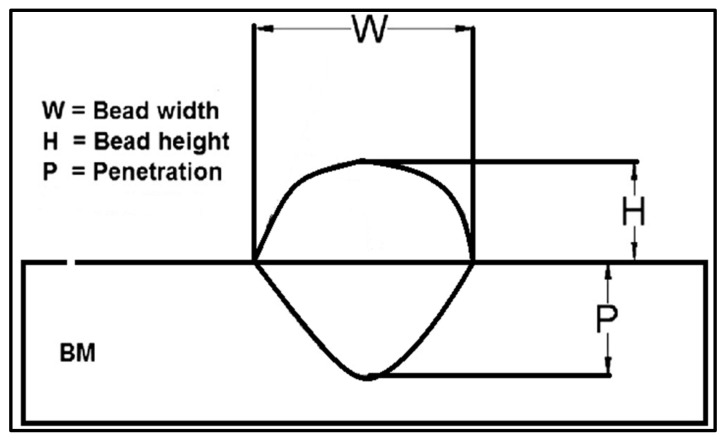
Bead geometry for V-shape butt joint.

Based on an extensive literature review, the selection of factors and their levels have been provided in Table 2. The bead geometry was optimized for further analysis of temperature and thermal stresses, as shown in Figure 2.

The bead geometry was divided into bead height, width, and penetration, which were measured using linear measuring instruments.

## 3. Experimental Design and Analysis

Taguchi design is an efficient, straightforward approach and saves more time by minimizing the number of experiments, as stated by Choudhury et al. [28]. Hence it is used in the present study. Table 3 shows the results of output responses concerning different sets of input factors. Nine experiments were performed by Taguchi orthogonal Array (L_9_3^3^(OA)). A grey relational analysis (GRA) was employed to find the optimal setting for a final geometrical model for further investigation by FEA. GRA is a multi-objective optimization technique in which various responses can be optimized simultaneously. Three input factors, i.e., rate of flow (F), welding current (I), and gap distance (G), were selected to study their influence on bead height and width (H and W) and area of penetration (P) of the weld. Grey’s relational coefficient was determined to find the correspondence in actual and theoretical results.

Two criteria correspond to the output, i.e., the smaller- and larger-the-better cases. In the present study, weld geometry comprises bead height (H) and bead width (W) that corresponds to the lower case that can be represented by Equation (1):

(1) $$x_{i}(k)=\frac{\max y_{i}(k)-y_{i}(k)}{\max y_{i}(k)-\min y_{i}(k)}$$

And similarly, the response variable, penetration (P), follows the larger-the-better case, as shown in Equation (2):

(2) $$x_{i}(k)=\frac{y_{i}(k)-\min y_{i}(k)}{\max y_{i}(k)-\min y_{i}(k)}$$

In the above expressions (Equations (1) and (2)), $x_{i}\left(k\right)$ represents the grey relation generated value, where $\min y_{i}\left(k\right)$ and $\max y_{i}\left(k\right)$ shows the lowest and highest values for all kth responses, respectively, whereas $x_{0}\left(k\right)$ represents the initial (ideal) sequence for output responses (k = 1–9). The final grey grades show the DOF between sequences $x_{0}\left(k\right)$ and $x_{i}\left(k\right)$. The grey relational coefficient, $\delta_{i}\left(k\right)$ was calculated using Equation (3):

(3) $$\delta_{i}(k)=\frac{\Delta_{\min}+\Psi \Delta_{\max}}{\Delta_{0i}(k)+\Psi \Delta_{\max}}$$

In Equation (3), $\Delta_{0i}\left(k\right)$ shows the deviation sequence of the reference, $x_{0\left(k\right)}^{*}$ and $\;x_{i\left(k\right)}^{*},$ i.e., the ideal and comparability sequences. The symbol Ψ represents the identification coefficient, and its value ranges from 0–1. Usually, it takes a value of 0.5 to ensure equal importance of all factors. The deviation sequence $\Delta_{0i}\left(k\right)$ is determined using Equation (4):

(4) Δ0i(k)=|x0(*k)−xi(*k)|

(5) $$y_{i}=\frac{1}{n}\sum_{k=1}^{n}\delta_{i}(k)$$

whereas in Equation (5), $y_{i\;}\mathrm{and}\;n$ show the final grey grades and response variables for corresponding experiments, respectively. Higher values of grey grades show better agreement between the ideal and given reference, i.e., $x_{0}\left(k\right)\;\mathrm{and}\;x_{i}\left(k\right).$ The reference sequence shows the better sequence; thus, the high value of grade corresponds to combinations of input parameters closer to the optimum for each experiment using L_9_3^3^(OA). Plots of the main effect and grand mean of grey relational grades are essential for efficient analysis.

## 4. Grey Relational Analysis

Responses are measured after performing experiments according to the design plan, as provided in Table 3. After experimentation and data collection, the sequences $x_{i}\left(k\right)\;\mathrm{and}\;x_{i\left(k\right)}^{*}$ are calculated using Equations (2) and (3) by keeping both cases in mind, i.e., smaller and larger-the-better cases, respectively. Similarly, the deviation sequence $\Delta_{0i}\left(k\right)$ was determined using Equation (4) and, $\Delta_{max}$ and $\Delta_{min}$ are the higher and lower values in deviation sequences in each response, respectively, hence $\Delta_{max}=1$ and $\Delta_{min}=0$. As discussed earlier, the Ψ value is 0.5, which means all factors have equal importance. In Equation (3), $\delta_{i}\left(k\right)$ i.e., the grey relational coefficient for each run was determined, as shown in Table 4. Grey grades, $y_{i}$ were determined by taking the average of grey coefficients using Equation (5). Table 4 shows that the second experiment gives the highest relational grade, which means it agrees with the theoretical results.

It has been concluded that multiple performance characteristics have been transformed into optimized grey grades. As it is an orthogonal experimental design, it is easy to determine the main effects of input parameters with different grey grades. For instance, the average grey grade value for gas flow rate (F) at the first, second, and third levels were computed by taking the mean value of grey grades from the 1st to 3rd, 4th to 6th, and 7th to 9th experiments, as presented in Table 5.

Similarly, the mean grey grades can be determined for the other factors, namely welding current and gap distance. The results for each factor at each level have been summarized in Table 5. As discussed earlier, product quality will be nearer to the optimum as the value of grey grades increases. Therefore, for adequate performance measures, higher grey grades are desirable. The optimal combination of the factors for optimum bead geometry is F1, A2, and G3, as presented in Table 5. The signal-to-noise ratios for each factorial combination relative to optimization use Equation (6), as provided in Table 6.

(6) $$S/N=-10\log\left[\frac{1}{n}\sum_{i=1}^{n}\frac{1}{y_{i}{}^{2}}\right]$$

**Figure 3:**
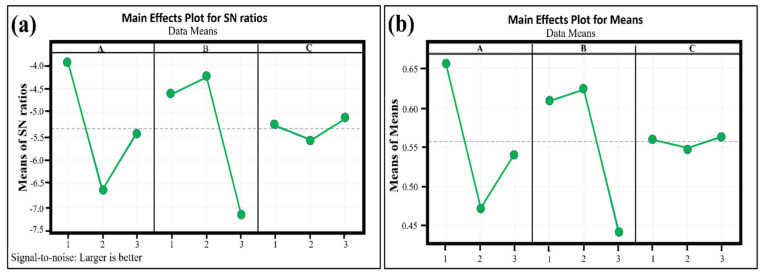
Results for verification of grey relational grades by signal-to-noise (SN) ratio (**a**) main effect plot for S/N ratio of process parameters (**b**) mean effect plot for grey relational grade. A: Gas Flow Rate, B: Welding Current, C: Gap Distance.

In the above equation, *n* is the number of experiments and $y_{i}$ shows the measured output response. Mean S/N ratios for the three factors at each level are computed and presented in Table 6. MINITAB 16 software was used to plot the parameters’ main effects, as shown in Figure 3. This figure illustrates the optimal condition of factors, i.e., F1, A2, and G3 (i.e., gas flow rate (F) = 10 L/min, welding current (I) = 80 A, and gap distance (G) = 2.5 mm), which is the same as obtained by grey relational analysis. 

## 5. Finite Element Analysis (FEA)

The primary purpose of the finite element analysis method is to determine the solutions to different engineering problems with complex geometries to reduce the calculations and saves time. Experimentation can be used to find unknown factors and optimal combinations, but it requires additional testing and samples to study each behavior. This will has a high cost and time is needed to prepare each model and procure the equipment. The FEA technique predicts the approximate solutions to save time and cost within an acceptable range. Depending upon the accuracy level of the model, the optimized structure or shape is integrated into the finite element analysis. Different boundary conditions must be satisfied according to the structure and field variables. The steps are generally categorized into three main stages: (a) pre-processing, (b) solution, and (c) post-processing. Pre-processing includes the geometric and material properties of the elements, e.g., dimensions, element types, meshing the model, loadings, and boundary constraints. In the solution, the model is built to find the unknown variables. In post-processing, results are evaluated and interpreted.

### 5.1. Implementation of FEA (Material, Modeling, and Properties)

**Figure 4:**
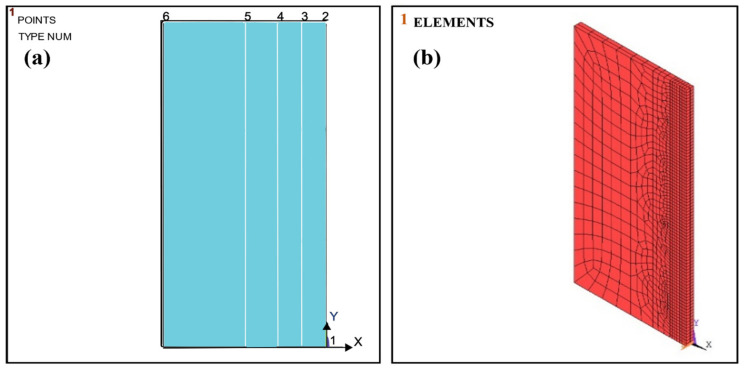
(**a**) Solid model. (**b**) Meshed model using ANSYS Mechanical APDL. 1. first pick point on welding area, 2. second pick point on welding area, 3. third pick point on welding area, 4. fourth pick point on welding area, 5. fifth pick point on welding area, 6. sixth pick point on welding area.

The present study analyzed the solid model using the finite element method (ANSYS APDL 2020R1) of the butt welded joint (mild steel). The model comprised two mild steel plates with dimensions 80 mm × 20 mm × 3 mm, welded at the ends using a V-shaped butt joint. The welded parts are modeled and analyzed to observe the temperature distribution and stresses induced during welding. The study was conducted by taking a single pass. Figure 4a,b shows the solid and meshed geometric models. The temperature at each node was adapted in the welding environment for the element types, PLANE 55 (2D), containing four nodes, and SOLID70 (3D) with a single DOF. Meshing was performed in four areas (mm) as A1 (0.0012), A2 (0.0025), A3 (0.005), and A4 (0.0065) for better estimation. Transient thermal analysis was first performed by providing heat flux concerning time to predict the temperature change. Then, stresses and distortion were obtained by coupling the thermal and static structural analyses. The mesh size used in both steps was the same. It was assumed that the base metals and welded regions have the same thermal properties. There is no penetration, and overfilled welds are considered for simplicity.

**Figure 5:**
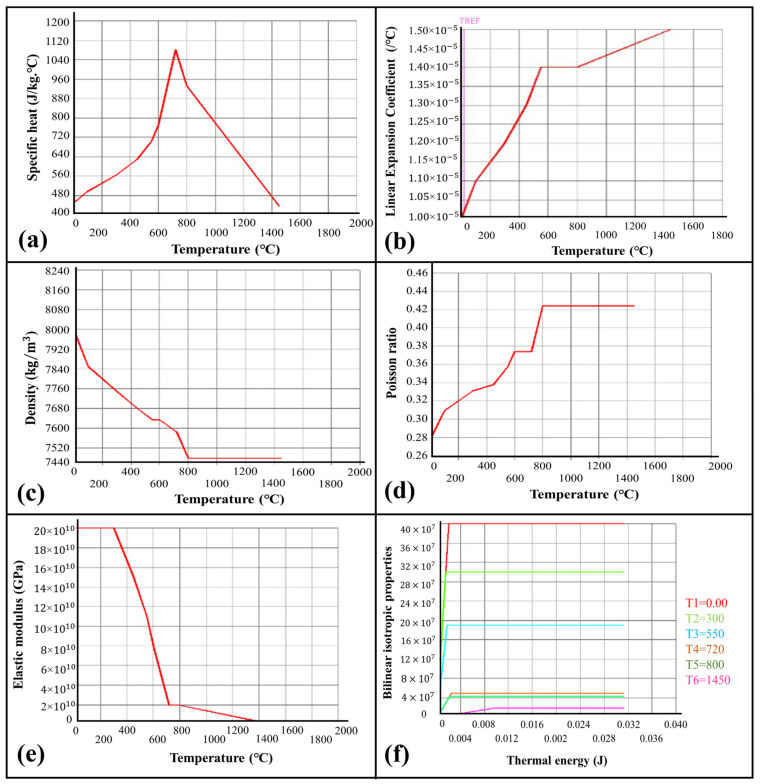
Temperature-dependent thermal properties of mild steel considered for the analysis. (**a**) Specific heat. (**b**) Linear expansion coefficient. (**c**) Density. (**d**) Poisson’s ratio. (**e**) Elastic modulus coefficient. (**f**) Bilinear isotropic properties.

The temperature-dependent properties, such as Poisson’s ratio, Young’s modulus, conductivity, density, and coefficient of thermal expansion, were characterized through differential scanning calorimetry and dynamic mechanical analysis, as illustrated in Figure 5a–f. The melting temperature of mild steel is 1450 °C.

### 5.2. Thermal Loading

**Figure 6:**
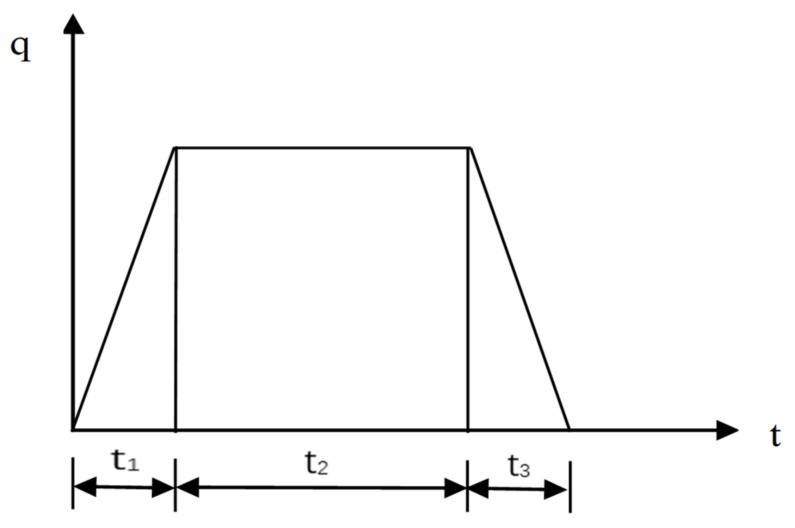
Load inputs function.

The heat flux varies with time after the thermal load is applied during thermal analysis. Figure 6 shows that the load inputs are provided in three steps (t1–t3), and the total time is up to 1100 s. For the first ten seconds, a ramp input is given, followed by a step input up to 100 s, and then again, a ramp input is given up to the end. When welding begins, the heat steadily increases until it reaches a particular value. For a short period, it remains stable (no change) and then decreases steadily until the temperature of the welded plates reaches room temperature.

The input heat was determined by the product of arc efficiency, provided voltage, and welding current, which are 0.7 V, 15 V, and 80 A, respectively, in the present study. The maximum heat flux determined is 0.62 × 10^6^ W/m^2^. The node solutions for temperature in this step were regarded as inputs into the stress analysis. This is because the residual stresses produced in heating and cooling periods result from temperature fields.

### 5.3. Boundary Conditions

The boundary conditions should be set for the thermal and static analysis, which can be applied to the plates for different parameters. The boundary conditions for other parameters used in this study are the following.

### 5.4. Thermal Analysis

**Figure 7:**
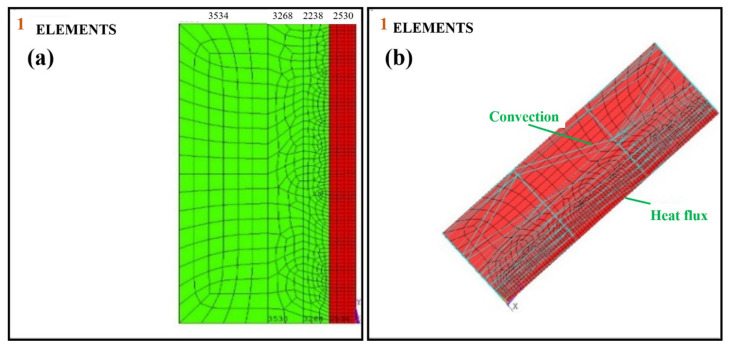
Boundary conditions applied to the thermal analysis. (**a**) number of elements in the constrained area (**b**) application of convection and heat flux to the plate area in symmetric model.

The convection heat was applied to the surface area of the plates, which are meshed in identical elements in the symmetric model, as illustrated in Figure 7. The Gaussian heat source was applied on surface areas A5 and A10.

The welded region where the plates are in contact is supposed to be insulated, i.e., no heat flow across this region. The value of the coefficient of convection is 20 W/m^2^ °C. The uniform temperature, 220 °C, was given to other remaining areas.

## 6. Static Analysis

After the thermal analysis of temperature distribution, the model is inserted into the static analysis, so new conditions should be applied. During static analysis, the degree of freedom should be zero to constrain the model in all directions.

## 7. Heat Transfer and Mechanical Analysis

Heat transfer and mechanical analysis is the first step in the FEA while running the TIG welding simulation using ANSYS. The following equation can describe the formulation in FEA for the elements:

(7) $$\left[C(T)\right]\left\{\hat{T}\right\}+\left[K(T)\right]\left\{T\right\}=\left\{Q(T)\right\}$$

The heat conduction equation must be integrated with regard to time for this study. The Crank–Nicolson/Euler theta integration method is used to solve these system equations. This element type can conduct heat in three dimensions. The heat conduction versus time equation must primarily be integrated into this kind of analysis. The heat is provided by the electrode as the heat flux and is considered input for heat transfer from the electrode to the plates based on setting factors and arc efficiency. The heat flux density at any point from the heat source center can be computed using the following equation:

(8) $$Q=Q_{m}e^{\frac{-3r^{2}}{R^{2}}}$$

(9) $$r^{2}={\left(X-0\right)}^{2}+{\left(Y-V\times Time\right)}^{2}$$

Since, at the y-axis, the value of *x* is zero, Equation (8) can be written as:

(10) $$Q=Q_{m}e^{\frac{-3(X^{2}+{\left(Y-VT\right)}^{2})}{R^{2}}}$$

where $Q_{m}$ is the maximum heat at the center of the heating source, sub-degree *R* is the effective radius of the heating source, and *r* is the distance between the center of the arc heating source and point A. The heat source model estimates temperature with finite element analysis in TIG welding. For stress analysis, heat transfer analysis was carried out to compute the temperature at the nodes as the time function, and then the structural analysis was performed using the temperatures attained from the heat analysis. The input values of the selected factors and constant factors used in the simulation are given in Table 7.

The constant voltage of 15 V was supplied to each experiment, and the selected value for welding current achieved from the grey relational analysis was 80 A. The value of total heat flux at the welded region was calculated using the following equation:

(11) $$q=\frac{Q}{A}$$

where *Q* is heating flux density, and *A* is the area of the welded region, which is the same for all the experiments. By computing the value of *Q* using the equation and the area of the welded triangular region (Q=840 W and $A=4.5\times 300\times 10^{-6}m^{2}$), the total heat flux was determined (*q* = 0.62 × 10^6^ W/m^2^). The FEA for the thermal and structured analysis of TIG welding using ANSYS Mechanical APDL works according to the following Equations:

(12) $$K\left(\frac{\partial^{2}T}{\partial x^{2}}+\frac{\partial^{2}T}{\partial y^{2}}+\frac{\partial^{2}T}{\partial z^{2}}\right)-q+h(T-T\dot{\alpha })=0$$

(13) [C]{T}={f}

(14) $$[K^{*}]\left\{q\right\}=\left\{F\right\}$$

(15) $$[K{]}^{*}=\frac{AE}{L}\left(\begin{array}{cc} 1 & -1\\ -1 & 1 \end{array}\right)$$

(16) $$F=\int B^{t}D\varepsilon_{0}\delta \theta$$

**Figure 8:**
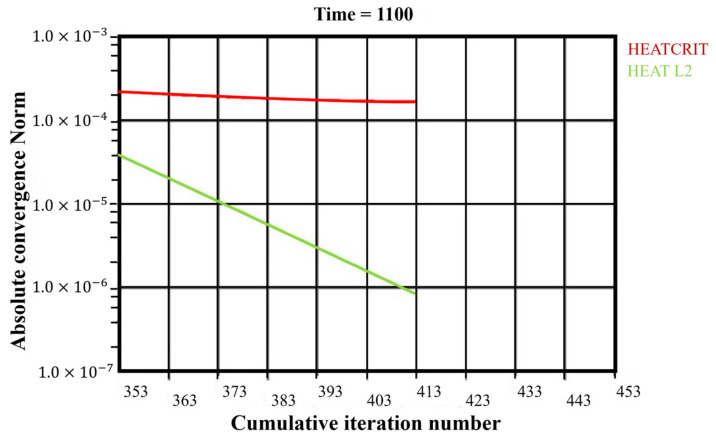
Convergence curve for thermal analysis.

The notations used in the above equations “SDPBS. http://sdpbs.math.uwm.edu/about.php (accessed on 22 December 2022)” are explained in Table 8. The total time to arrive at the solution for temperature was 1100 s, illustrated in Figure 8, and the number of sub-steps in each iteration was 50. The x-axis and y-axis are labeled as cumulative iteration numbers and absolute convergence norms.

The absolute convergence norm uses normalized values to quickly solve the variables using the Newton–Raphson method. A solution within the range means the function value should be higher than the value of the vector norm of the function for each sub-step. It is observed that the HEAT CRIT values are higher than the HEAT L2 (vector norm) at every sub-step, so it can be concluded that the solution is within tolerance and is correctly converged.

## 8. Results and Discussion

### 8.1. Regression Model for Grey Relational Grades and Corresponding Response Variable

The quadratic regression model was employed to analyze the grey relational grades for optimizing the corresponding bead height, width, and penetration. The regression model for an overall grey grade is expressed by Equation (18):

(17) $$\begin{array}{l} GRG=0.5917-0.0607F-0.0007I-0.0107G\\ -0.0247FI+0.1673FG-0.0077IG+0.1907F^{2}\\ -0.1833I^{2}+G^{2} \end{array}$$

The mathematical models predict the response variables (bead height, bead width, and penetration) for the given input parameters to the grey relational grades given by the Equations (19)–(21). A two-factor interaction model was suggested to predict the actual bead height, bead width, and penetration:

(18) $$\begin{array}{l} H=-0.0857+0.0573F+0.01401I-0.7404G\\ -0.0015FI+0.0334FG+0.0034IG\\ -0.1833I^{2}+G^{2} \end{array}$$

(19) $$\begin{array}{l} W=+1.8961+0.3428F+0.04298I-0.1970G\\ -0.0021FI-0.0716FG+0.00211IG \end{array}$$

(20) $$\begin{array}{l} P=+5.8028-0.1879F+0.01389I-4.2446G\\ -0.00328FI+0.02321FG+0.01587IG \end{array}$$

In the above equations, the terms GRG, H, W, and P show the grey grades, bead height, width, and penetration, respectively. The parameters F, I, and G are the gas flow rate, welding current, and gas distance, respectively. Statistical optimum value for grey relational grade obtained for the welding current range is 61 to 83 (A), and the gas flow rate is up to 1.79 (L/min). According to the optimum results achieved from grey relational analysis, simulation of the TIG welding has been performed on ANSYS APDL 2020R1 using finite element analysis to analyze the temperature field and thermal stresses induced. Autodesk Inventor carried out the welding and then imported it to the ANSYS workbench for further research. The following results have been achieved for temperature and stress analysis. First, the temperature variations over time and thermal stresses for the optimum geometry are studied. ANSYS APDL was used to generate the model, and finite element analysis (FEA) was employed to predict the responses. Second, a transient analysis followed by the coupled field analysis was performed to predict temperature variations and thermal stresses. Finally, the voltage, efficiency, and welding variation on the temperature field are presented.

### 8.2. Temperature Analysis

**Figure 9:**
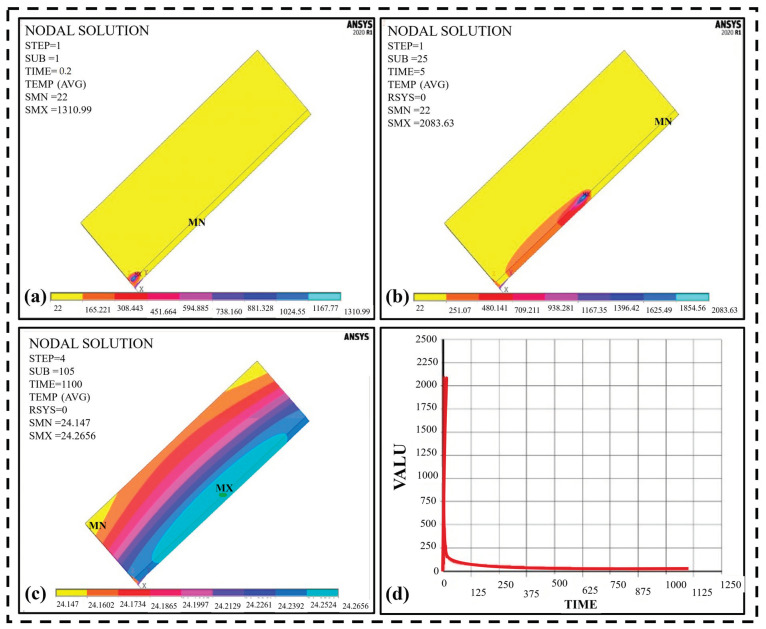
Temperature distribution at time t at subsequent steps: (**a**) 1st pick (beginning of weld) (**b**) 25th pick (**c**) after the weld pass (cooling) (**d**) Variation of temperature with the time for 1100 s (one complete pass).

Based on the simulation and analysis of the optimized weld bead geometry of the model, the temperature field data from the thermal analysis are loaded into the stress analysis. Figure 9a shows the start of the TIG welding to move the torch at the first subsequent step.

The minimum temperature is equal to the uniform temperature (22 °C), and the maximum temperature provided to melt the boundary for welding both the plates is 1310.99 °C for V = 0.01 mm/s at the first point of a welded region, which is less than the melting temperature (1450 °C). The maximum temperature observed in the welded area at the last pick is 2083.63 °C which exceeds the melting temperature of mild steel (1450 °C), as shown in Figure 9b. This depicts the temperature at the weld region subject to the boundary conditions. The maximum temperature should be at the flux area. It also shows that the temperature drops quickly from the boundary to the central region and is distributed through the transverse and longitudinal axis, as provided in Figure 9c. The thermal area also changes as the heat source moves along the path and the pool that melts moves with the heat source. The maximum temperature drops to 24.26 °C and becomes nearly equal to the uniform/room temperature, showing that the plates quickly cool down after welding.

This occurs in the cooling cycle, which shows a slight decrease in temperature after the thermal phase until it reaches room temperature. Figure 9d describes the temperature variation with the time away from the heat zone. This curve is achieved by selecting the nearest node to the welded region. It depicts the temperature change with time and distance. When a certain amount of heat is applied, the temperature gradually increases to the peak value during the first phase, then drops to room temperature at a constant rate, called a cooling cycle. It is clear from the graph that the peak temperature recorded for the given time of 1100 s is 2083.63 °C on the y-axis. For a small range of the x-axis, i.e., 0–20 s, it can be observed that the peak temperature is reached very quickly, but as the torch moves further, the temperature is distributed uniformly till the end of the pass. Then the cooling cycle takes place to achieve its room temperature.

The results of heating are quite similar to Liu et al. (2022) [29] and Nie et al. (2020) [30] for aluminum, which showed the reliability of our conclusions.

### 8.3. Stress Analysis

**Figure 10:**
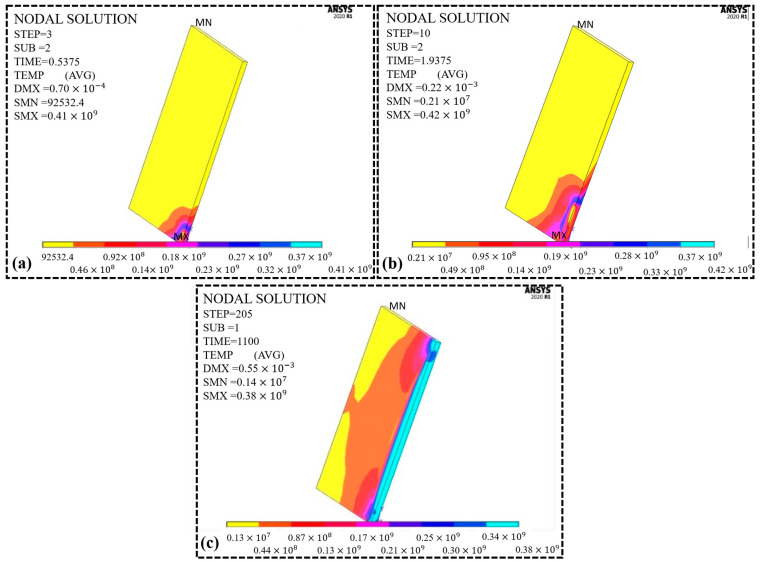
Thermal stress distribution (**a**) 1st pick (**b**) last pick (complete pass) (**c**) after the weld pass (cooling).

Prediction of thermal stresses is essential to minimize the failure within welded plates of mild steel, which causes the failure of the welds. Thermal stresses result from thermal analysis in the subsequent process, as shown in Figure 10a–c. At the beginning of the welding process, the tensile thermal stresses are developed but then turn into compressive thermal stresses upon solidification (cooling). When the heat source moves away, the parts are cooled down, and the atoms are compressed, resulting in nodes being compressed. Figure 10a shows the beginning of the welding process when the heat source starts to melt the material, which indicates that the maximum thermal stress recorded is 416 MPa at the third step. As the heat source moves further (STEP-10), the thermal stress increases and reaches 424 MPa, as presented in Figure 10b. Finally, when the first pass was completed (STEP-205, TIME-1100s), thermal stresses decreased and dropped to 388 MPa, as presented in Figure 10c. These changes are associated with temperature variations, which correctly describe the process [30].

**Figure 11:**
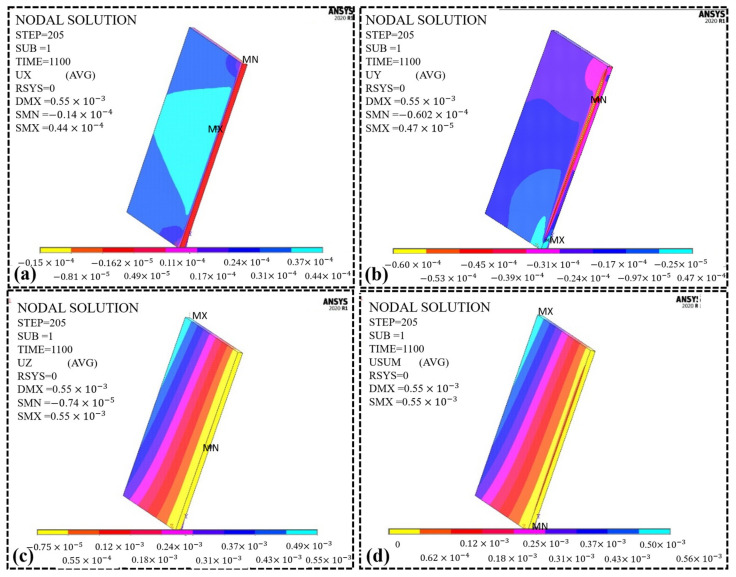
Deformation along with the three-axis (**a**) along the x-axis in the welding direction, (**b**) along the y-axis transverse to the welding direction, (**c**) along the z-axis through the thickness of the plates, and (**d**) vector sum of all the components.

The thermal stresses increase due to undergoing early heating and cooling mechanisms, which results in thermal gradients and associated thermal stresses. Thermal gradients decrease as the temperature increases, which results in higher thermal stresses. As the process repeats in TIG welding, thermal stress may be produced inside the parts. Heat treatment can minimize this issue before other operations, such as surface cleaning, finishing, welding, etc. The thermal stress will affect the welding quality of the mild steel parts due to deflection. The pre-heating mechanism will increase the material’s ductility, which improves the chances of relieving the thermal stresses. It will produce deformation in different directions to minimize thermal stresses, i.e., the x-axis, y-axis, and z-axis. Figure 11a shows the deformation in the x-direction along the path of the heat source. This is due to the thermal/residual stresses, mainly resulting from temperature variations. The maximum deformation recorded along the direction of the heat source is 0.440 × 10^−4^ m (0.044 mm).

Similarly, the maximum deformation along the y-axis is 0.0047 mm, which is the transverse direction to the direction of the heat source, as shown in Figure 11b. The maximum deformation is demonstrated at the welding start due to the maximum stress in this region. As the heat source moves linearly along the x-axis, less deformation occurs as there is no movement along the y-axis. The maximum stress is distributed uniformly in the central region, shown by the red area. Figure 11c shows the deformation along the z-axis indicated by the plates’ thickness. The maximum deformation recorded along the thickness of the plates is 0.556 mm. The resultant deformation is the vector sum of all the components, as shown in Figure 11d. The maximum resultant deformation recorded in the plates is 0.558 mm. The main reason for induced deformation is the thermal stresses resulting from temperature variations. The more uniform the stress is distributed, the less deformation will be induced in the plates.

### 8.4. Effect of Welding Parameters on Temperature Field

After finite element analysis of TIG welding of an optimized bead geometry, it is necessary to determine the effect of parameters on temperature to minimize the thermal stresses in welding. In the present analysis, the parameters considered for investigation vary in voltage, efficiency, and welding speed. The thermal stresses are produced due to temperature variations caused by the process parameters, so the temperature needs to be investigated. The results are summarized in Table 9.

### 8.5. Effect of Variations in Voltage on Temperature

**Figure 12:**
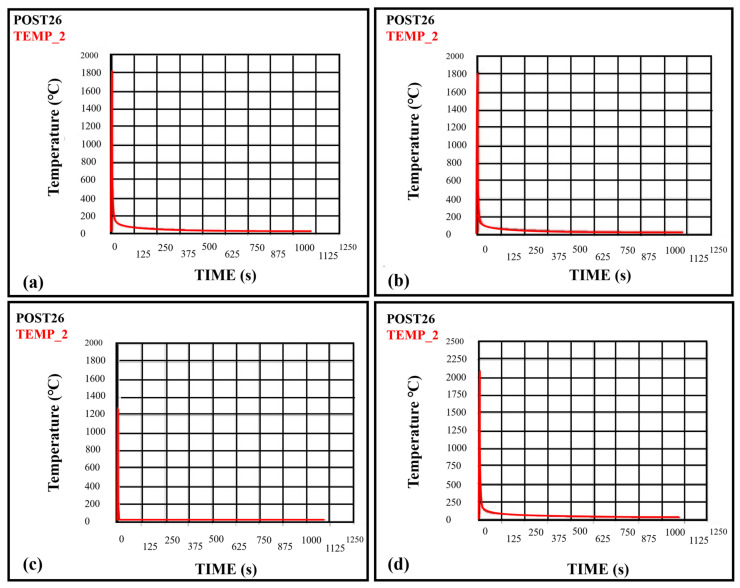
Temperature variation with time at the weld zone (**a**) for 12 Volts (**b**) for 13 Volts (**c**) for 14 Volts (**d**) for 15 Volts.

The change in voltage will lead to temperature variation, resulting in thermal stresses. The values taken for voltage are 12 V, 13 V, 14 V, and 15 V, and keep other parameters constant, are shown in Figure 12a–d. It can be observed from the figure that as the voltage increases from 12 V–15 V, the temperature also increases from 1362.22 °C to 2083.63 °C. The peak temperatures for voltages of 12, 13, 14, and 15 volts are 1362.22 °C, 1771.05 °C, 1926.80 °C, and 2083.63 °C, respectively. Therefore, it is concluded that the temperature increases with the voltage increase. The results were also verified by comparing with Ghazvinloo et al. [31], which showed that voltage significantly impacts the temperature, which will further affect fatigue life, energy, and bead penetration.

### 8.6. Effect of Variations in Efficiency on Temperature

**Figure 13:**
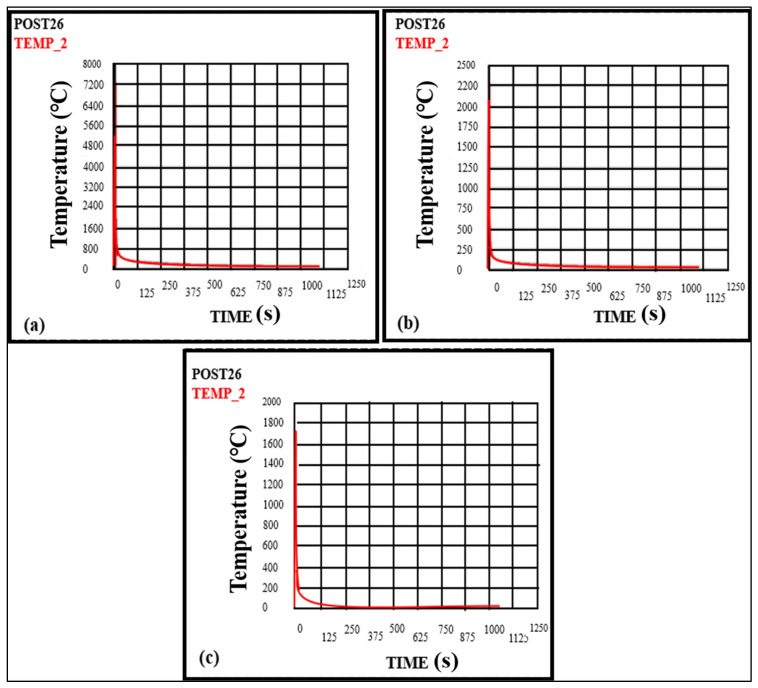
Temperature variation with time at the weld zone (**a**) for 0.6 Efficiency (**b**) for 0.7 Efficiency (**c**) for 0.8 Efficiency.

The efficiency variation will also change temperature, further leading to thermal stresses. The efficiencies taken in this study are 0.60, 0.70, and 0.80, and other parameters are kept constant, as shown in Figure 13a–c. It can be determined from the figure that the temperatures at the weld zone for efficiencies of 0.60, 0.70, and 0.80 are 1750.01 °C, 2083.63 °C, and 4001.78 °C, respectively. Therefore, it is concluded that as the efficiency of the weld joint increases, the peak temperature also increases [32]. Arc efficiency measures how effectively the electric arc is used during TIG welding. It is calculated by comparing the amount of heat generated by the arc to the amount of electrical energy consumed. To calculate the arc efficiency (*E*) during TIG welding, arc voltage (*V*), arc current (*I*), welding speed (*v*), and electrode efficiency (*E_electrode_*). The formula for efficiency is:

(21) $$E=\left[\frac{H_{i}}{E_{i}}\right]\times 100\%$$

(22) $$\mathrm{where},\;\left\{\begin{array}{l} H_{i}=V\times I\times E_{electrode}\times 60\\ E_{i}=V\times I\times 60 \end{array}\right\}$$

This is a factor that takes into account how efficiently the electrode is transferring welding current to the workpiece. The electrode efficiency for TIG welding is typically around 70%. Note that voltage, welding current, and welding speed values should be measured during the welding process. The electrode efficiency value is an estimate based on the type of electrode being used.

### 8.7. Effect of Variation in Welding Speed on Temperature

**Figure 14:**
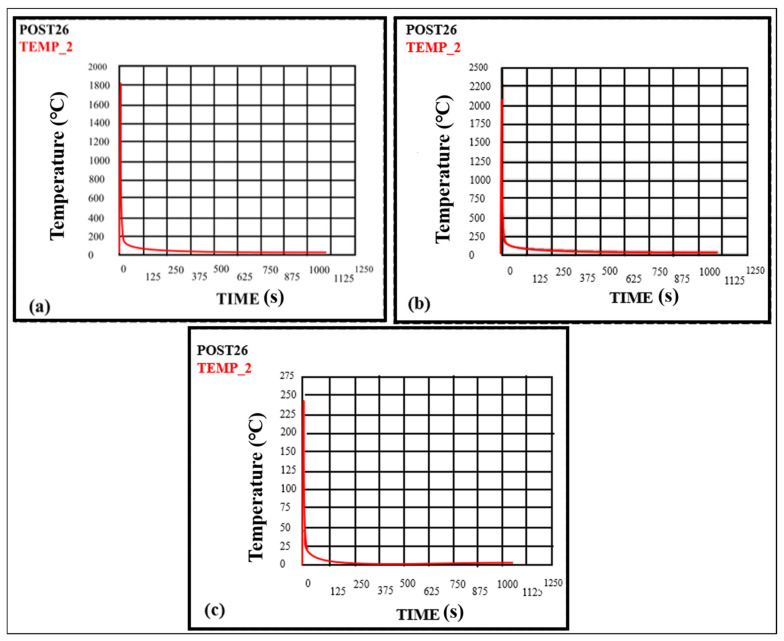
Temperature variation with time at the weld zone (**a**) for v = 0.01 mm/s (**b**) for v = 0.02 mm/s (**c**) for v = 0.03 mm/s.

The change in welding speed also affects the temperature field, which will cause thermal stresses and anisotropy in the weld pool. The temperature variation is also studied for the welding speed of 0.01, 0.02, and 0.03 mm/sec, as shown in Figure 14a–c. The temperatures obtained for the welding speed of 0.01, 0.02, and 0.03 mm/sec are 2083.63 °C, 1329.57 °C, and 243.78 °C, respectively. It is concluded that the temperature decreases with the increase in welding speed and reaches its lowest value. In a way, the temperature field can be optimized by varying the welding parameters. Welding speed also significantly influences the microstructure and mechanical properties of the joining due to the variation of temperature field, which is also stated by Cevik et al. (2021) [33].

#### Confirmation Test for Optimization

After selecting optimal parameters, it is necessary to predict and verify the responses of the welding process with an optimum combination of process parameters. Firstly, the developed models for all the responses were validated through six additional runs. The values for input models (other than the selected design) lie in the defined ranges taken for additional confirmatory runs. The final calculations and results are summarized in Table 10. The percent error for each predicted and experimental response for comparison was determined by Equation (21) [34]. A percent error of less than 5% will be in reasonable agreement to predict the bead height, bead width, and penetration with reasonable accuracy. Therefore, it can be observed from the table that all the predicted and experimental results are in good agreement and validate the developed models. The predictable error in grey relational grade (*ŷ*) is calculated by optimum levels of input process parameters using Equation (22) [35] as:

(23) $$Error(\%)=|\frac{Experimental-Predicted}{Predicted}|\times 100$$

(24) $$\hat{y}=y_{m}+\sum \left(\bar{y}-y_{m}\right)$$

In the above expression, $y_{m}$ represents the total mean value of the grey relational grade, $\bar{y_{i}}$ shows the grade value at an optimum level, and ŷ represents the predicted value of the grey relational grade. Table 11 compares the predicted values with the actual values of bead geometry using optimal welding parameter combinations.

In this research, performance characteristics have been selected with the parameters of bead geometry and given a weightage equal to all responses. The results show that using the optimum setting of process parameters (F1, A2, and G3) caused higher penetration and less bead height and bead width.

## 9. Conclusions

In this study, we utilized the Taguchi-grey relational analysis approach to optimize the bead geometry in TIG welding. We also conducted numerical simulations using finite element analysis to analyze temperature and stress variations. Our results showed that the welding current was the most influential factor affecting the performance measures, followed by the flow rate. We found that the optimal values to optimize the bead geometry were a heat flux of 0.62 × 10^6^ W/m^2^, a gas flow rate of 10 L/min, a welding current of 80 A, and a gap distance of 2.5 mm. The maximum deformations induced by the thermal stress along the x-axis, y-axis, and z-axis were 0.044 mm, 0.0047 mm, and 0.556 mm, respectively. Less deformation occurred along the y-direction as there was no motion of the heat source. By changing the voltage, efficiency, and welding speed, we observed that the temperature field increased with voltage and efficiency but decreased by increasing the welding speed of the torch. Our models were validated through additional experiments.

Overall, this study demonstrates the potential of using Taguchi-grey relational and finite element analyses to optimize the bead geometry in TIG welding. By achieving uniform temperature and stress distribution, we can improve the mechanical properties and quality of the weld while saving resources and reducing costs. In future studies, we can consider heat dissipation or losses and multiple passes for even more precise modeling.

## Figures and Tables

**Table 1:** Mild steel composition.

C (%)	Si (%)	Mn (%)	S (%)	P (%)
0.05–0.25	<0.35	0.30–0.90	<0.05	<0.05

**Table 2:** Factors and their levels.

Input Parameter	Units	Levels		
		Low	Middle	High
A: Gas Flow Rate	L/min	10	12	14
B: Welding Current	A	60	80	100
C: Gap Distance	mm	1.5	2.0	2.5

**Table 3:** Design matrix.

Input Factors				Output Factors		
				Smaller-the-Better		Larger-the-Better
Run	F: Gas Flow Rate	I: Welding Current	G: Gap Distance	H: Bead Height (mm)	W: Bead Width (mm)	P: Penetration (mm)
1	10	60	1.5	0.136	5.535	1.344
2	10	80	2.0	0.093	4.456	1.029
3	10	100	2.5	0.267	6.324	1.204
4	12	60	2.0	0.183	6.829	1.045
5	12	80	2.5	0.129	5.232	1.079
6	12	100	1.5	0.263	7.304	1.234
7	14	60	2.5	0.129	4.690	1.127
8	14	80	1.5	0.143	5.640	1.006
9	14	100	2.0	0.188	6.339	1.137

**Table 4:** Ranking of Grey Relational Grades in the optimization process.

Run No.	Grey-Coefficient			Grey Grades	Ranking
	Bead Height $\delta_{i}\left(1\right)$	Bead Width $\delta_{i}\left(2\right)$	Penetration $\delta_{i}\left(3\right)$		
1	0.669	0.569	1.000	0.746	2
2	1.000	1.000	0.349	0.783	1
3	0.333	0.433	0.547	0.438	7
4	0.492	0.375	0.361	0.409	9
5	0.707	0.647	0.389	0.581	4
6	0.339	0.333	0.606	0.426	8
7	0.707	0.859	0.438	0.668	3
8	0.635	0.546	0.333	0.505	5
9	0.478	0.431	0.449	0.453	6

**Table 5:** Mean grey grades.

Factors	Grey Relational Grades			Main Effect	Rank
	Level 1	Level 2	Level 3		
Gas Flow Rate	0.656 *	0.472	0.542	0.183	2
Welding current	0.608	0.623 *	0.439	0.184	1
Gap Distance	0.559	0.548	0.562 *	0.014	3

* Optimum levels (Mean grey relational grades ($y_{m})=0.5566$).

**Table 6:** Mean responses.

Factors	Mean S/N Response Values				
	Level 1	Level 2	Level 3	Difference	Rank
Gas Flow Rate	−3.949	−6.629	−5.442	2.680	2
Welding Current	−4.603	−4.258	−7.159	2.901	1
Gap Distance	−5.299	−5.590	−5.131	0.458	3

Note: The total mean OF s/n ratio is −5.345.

**Table 7:** Input parameters for simulation.

Weldment Length (L) (mm)	Weldment Width (W) (mm)	Weldment Height (H) (mm)	Welding Voltage (U), Volts	Welding Current (I), Amp	Welding Speed (V), mm/sec	Welding Thermal Efficiency (YITA)	Effective Heat arc Rad© (R), (mm)
0.1	4.456	0.093	15	80	0.01	0.7	0.003

**Table 8:** Notations and their explanation.

Symbol	Meaning	Symbol	Meaning
K	Thermal conductivity	A	Welding area
T	Initial temperature	E	Young’s modulus
X	Plate width	F	Force function in structural
Y	Plate length	L	Length of material
Z	Plate thickness	[B]	Strain displacement matrix
q	Heat flux (Displacement function)	[D]	Elasticity matrix
h	Coefficient of heat transfer	f	Force function in thermal
$T\overset{`}{\alpha }$	Exposed temperature	[K]	Stiffness matrix
C	Shape matrix		

**Table 9:** Variations in temperature with the welding parameters.

Variables		Welding Current, A	Welding Area, mm^2^	Heat Flux, W/m^2^	Other Parameters		Temperature
Voltage, U (volts)	12	80	1350	0.48 × 10^6^	E = 0.7	V = 0.01	1362.22
	13	80	1350	0.52 × 10^6^	E = 0.7	V = 0.01	1771.05
	14	80	1350	0.57 × 10^6^	E = 0.7	V = 0.01	1926.80
	15	80	1350	0.62 × 10^6^	E = 0.7	V = 0.01	2083.63
Efficiency, E (%)	0.6	80	1350	0.53 × 10^6^	U = 15	V = 0.01	1750.01
	0.7	80	1350	0.62 × 10^6^	U = 15	V = 0.01	2083.63
	0.8	80	1350	0.71 × 10^6^	U = 15	V = 0,01	4001.78
Welding Speed, V (mm/sec)	0.01	80	1350	0.62 × 10^6^	U = 15	E = 0.7	2083.63
	0.02	80	1350	0.62 × 10^6^	U = 15	E = 0.7	1329.57
	0.03	80	1350	0.62 × 10^6^	U = 15	E = 0.7	243.78

**Table 10:** Comparison of Experimental and Predicted Results.

Runs	Input Variables					Response Variables		
	Gas Flow Rate	Welding Current	Gap Distance			Bead Height	Bead Width	Penetration
1	13	90	1.8	Experimental		0.187	6.090	1.125
				Predicted	GRD = 0.514	0.191	6.230	1.138
				Error		2.094	2.241	1.142
2	13	90	2.3	Experimental		0.197	5.930	1.251
				Predicted	GRD = 0.583	0.193	5.790	1.239
				Error		2.073	2.418	0.968
3	11	70	2.3	Experimental		0.08	5.217	0.890
				Predicted	GRD = 0.550	0.0791	5.303	0.850
				Error		1.138	1.622	4.705
4	11	70	1.8	Experimental		0.141	5.568	1.090
				Predicted	GRD = 0.639	0.145	5.692	1.140
				Error		2.759	2.178	4.385
5	13	70	1.8	Experimental		0.195	5.870	1.168
				Predicted	GRD = 0.556	0.191	5.810	1.137
				Error		2.094	1.033	2.726
6	11	90	2.3	Experimental		0.178	5.989	1.197
				Predicted	GRD = 0.524	0.173	5.836	1.141
				Error		2.890	2.622	4.907

**Table 11:** Confirmation Test Results.

Process Parameters Level	Initial Factor Setting	Optimal Factor Setting	
		Predicted Optimal Factors Setting	Experimental Optimal Factors Setting
	F1, A1, G1	F1, A2, G3	F1, A2, G3
Bead height (BH)	0.136		0.106
Bead width (W)	5.535		5.728
Penetration(P)	1.344		1.938
S/N ratio for overall GRD	−3.17	−2.65	−1.78
Overall Grey GRD	0.69	0.74	0.83

Improvement in S/N ratio 1.39, Improvement in grey relational grade 0.14.

## Data Availability

The data presented in this study is available on request from the corresponding author.
